# Influence of *STAT4* Genetic Variants and Serum Levels on Multiple Sclerosis Occurrence in the Lithuanian Population

**DOI:** 10.3390/jcm13082385

**Published:** 2024-04-19

**Authors:** Akvile Bruzaite, Greta Gedvilaite, Renata Balnyte, Loresa Kriauciuniene, Rasa Liutkeviciene

**Affiliations:** 1 Ophthalmology Laboratory, Neuroscience Institute, Medical Academy, Lithuanian University of Health Sciences, Eiveniu Street 2, LT-50161 Kaunas, Lithuania; greta.gedvilaite@lsmu.lt (G.G.); loresa.kriauciuniene@lsmu.lt (L.K.); rasa.liutkeviciene@lsmu.lt (R.L.); 2 Department of Neurology, Medical Academy, Lithuanian University of Health Sciences, Eiveniu Street 2, LT-50161 Kaunas, Lithuania; renata.balnyte@lsmu.lt

**Keywords:** *STAT4*, rs10181656, rs7574865, rs7601754, rs10168266, STAT4, serum levels, multiple sclerosis

## Abstract

**Background:** Multiple sclerosis (MS) is an autoimmune disease involving demyelination, inflammation, gliosis, and the loss of neurons. MS is a growing global health problem most likely caused by genetic, immunological, and environmental factors. However, the exact etiology of the disease is still unknown. Since MS is related to a dysregulation of the immune system, it could be linked to signal transducer and activator of transcription 4 (STAT4). To fully comprehend the significance of the *STAT4* gene and STAT4 serum levels in MS, further research is required. **Methods:** A total of 200 MS patients and 200 healthy controls participated in the study. Deoxyribonucleic acid (DNA) was extracted using silica-based membrane technology. Polymerase chain reaction was used in real time for genotyping. Using the ELISA technique, serum levels were measured. **Results:** *STAT4* rs7601754 AA genotype and the A allele were statistically significantly less frequent in MS patients (*p* = 0.003). Also, rs7601754 was associated with 1.9-fold increased odds of MS occurrence (*p* = 0.004). The rs7601754 AG genotype was more common in males with MS (*p* = 0.011) and was associated with 2.5-fold increased odds of MS occurrence in males (*p* = 0.012). STAT4 serum levels were statistically significantly lower in MS patients compared to the control group (*p* = 0.007). **Conclusions:** *STAT4* rs7601754 increases the odds of MS occurrence. STAT4 serum levels were statistically significantly lower in MS patients compared to the control group.

## 1. Introduction

Multiple sclerosis (MS) is an autoimmune disorder that impacts the central nervous system (CNS) and is characterized by gliosis, demyelination, the inflammation process, and the degeneration of nerve cells [1]. The accumulation of demyelinating lesions in the grey and white matter of the brain/spinal cord is the pathological hallmark of MS [2]. Young adults with MS, typically between the ages of 20 and 30, present with unilateral optic neuritis, partial myelitis, sensory abnormalities, or brainstem syndromes such as internuclear ophthalmoplegia. Worldwide, between 5 and 300 cases of MS per 100,000 people are reported, with a higher incidence in higher latitudes. The overall life expectancy is shorter than the population average (75.9 years vs. 83.4 years), and the risk of developing MS is higher in females than in males (approximately a 3:1 distribution between the genders) [3]. An autoimmune process has long been hypothesized as a mediating factor in MS. Research on experimental autoimmune encephalomyelitis (EAE), an animal model for MS, has suggested a crucial role for T helper lymphocytes. Researchers have studied how activated T cell subtypes contribute to the pathogenesis of MS, focusing on the genetic factors linked to the major histocompatibility complex (MHC) class II locus and the inflammatory response in the affected area [4]. Also, serum levels of interleukin-12 (IL-2), interleukin-4 (IL-4), interleukin-6 (IL-6), interleukin-13 (IL-13), interleukin-17 (IL-17), interleukin-21 (IL-21), interleukin-22 (IL-22), and interleukin-33 (IL-33) tend to be higher in MS patients in the active disease phase than in healthy controls and patients in remission, although interleukin-10 (IL-10) seems to help slow the disease’s progression. Moreover, certain gene variants of interleukin-2 receptor (IL-2R), IL-4, IL-6, IL-13, and IL-22 have been linked to the development of MS [5].

MS is defined as an immune system malfunction resulting in immune cells infiltrating the CNS [6]. After being activated outside the CNS, autoreactive T cells cross the blood–brain barrier (BBB) and are reactivated by nearby antigen-presenting cells. The release of proinflammatory cytokines activates microglia and astrocytes, attracts further inflammatory cells, and induces plasma cells to produce antibodies. This inflammatory process ultimately damages the tissue within the plaque [7].

**Figure 1:**
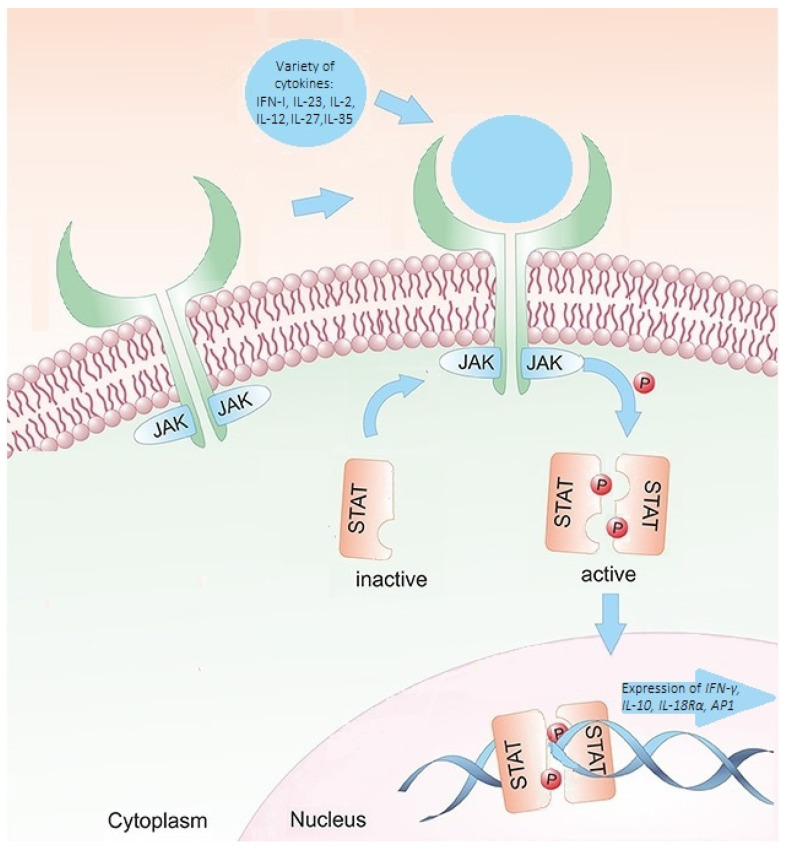
The JAK/STAT signaling pathway. Signals from extracellular cytokines are transmitted to the cell nucleus via the JAK/STAT signaling pathway. The transmembrane receptor of a cytokine binds to it and activates receptor associated JAKs, which phosphorylate STAT proteins. The transcription of the target genes is modulated by activated STAT proteins, which migrate into the cell nucleus as homo- or heterodimers [12,13].

MS and signal transducer and activator of transcription 4 (STAT4) may be related since MS has been linked to immune system dysfunction [6]. Janus kinases (JAKs) are the proteins through which members of class I and class II cytokine receptor families deliver their signals. Activated JAKs phosphorylate the STATs. After phosphorylation, the STAT proteins undergo cytoplasmic dimerization before migrating to the nucleus, where they bind to deoxyribonucleic acid (DNA) regulatory elements and initiate gene transcription. The STAT signaling cascade is highly selective. A specific subset of genes dependent on STAT proteins is transcribed by any cytokine or combination of cytokines that exerts an effect [8,9]. Consequently, a variation in STAT4 expression or activity might impact the regular immune system’s response and function, resulting in immunosuppression or autoimmune disorders. STAT4 is a crucial modulator of the immunological response (Figure 1) [8]. In addition, the *STAT4* gene is responsible for relaying signals from interleukin-12 (IL-12), interleukin-23 (IL-23), and interferon type 1 (INF-1) in T cells and monocytes. These signals ultimately lead to the differentiation of type 1 T helper cells and type 17 T helper cells, monocyte activation, and the production of interferon-gamma (IFN-γ) [10]. It is hypothesized that *STAT4* variants may influence the occurrence and function of immune cells involved in the pathogenesis of MS [11].

It is important to note that genetic factors alone cannot explain the occurrence of MS, as environmental factors also play a significant role in the development of the disorder [6,14]. In addition, a positive family history increases the risk of MS for siblings of affected patients by around 30% compared to the general population. More than 200 genetic loci have been linked to MS by genome-wide association study (GWAS) [15]. The epidemiology of MS suggests that smoking, low serum vitamin D levels, childhood obesity, and Epstein–Barr virus infection may contribute to the onset of the disease [16]. Research on the connection between genetic and environmental factors in MS is ongoing to develop new prevention and therapeutic strategies. Overall, the link between the *STAT4* gene and MS suggests that dysregulation of the immune system plays a significant role in disease development [6]. The basis of traditional MS treatment is immunomodulatory and anti-inflammatory medications. However, these measures cannot stop the degeneration of the nerve tissue. Neurologists should be aware of the latest findings on the development, pathophysiology, diagnosis, and treatment of MS [17]. Further research is essential to fully clarify the role of the *STAT4* gene and STAT4 serum levels in MS and ascertain whether focusing on this gene could be an effective treatment strategy.

## 2. Materials and Methods

### 2.1. Patients and Ethical Requirements

This research was authorized by the Kaunas Regional Biomedical Research Ethics Committee at the Lithuanian University of Health Sciences (LUHS) (No. BE-2-/61, approval date: 11 October 2017) and adhered to the Declaration of Helsinki’s criteria. The objective and procedure of the study were explained to each participant. Before participating, all 400 study individuals gave their written informed consent. The MS group was formed with 200 individuals. Criteria for inclusion in the MS group:

- Patients diagnosed with MS. The diagnosis of MS was confirmed using the 2017 diagnostic criteria, which include positive oligoclonal bands, typical demyelinating lesions on brain/spinal cord magnetic resonance imaging (MRI) scans (per the Magnetic Resonance Imaging in MS (MAGNIMS) criteria), and clinical symptoms/relapses [18,19].
- Males and females aged between 18 and 99 years.

Exclusion criteria for the MS group:

- Patients younger than 18 years.
- The patient has received a transfusion of blood or blood components within the last four weeks.
- The patient has received treatment with growth factors that counteract blood production in the last four weeks.

The control group included 200 patients. The control group comprised healthy individuals who matched the age and gender distribution of the MS group and who attended LSMUL, KK, the Neurology Clinic, and the Eye Clinic for a preventive examination. Criteria for inclusion in the control group:

- Healthy subjects without MS.
- Males and females aged between 18 and 99 years.

Exclusion criteria for the control group:

- Patients with subjective neurological complaints.
- Patients having spinal anesthesia.
- Patients with other neurological diseases without abnormalities in the demyelinating disorder of the brain and/or spinal cord.

After the subject groups were formed, the single-nucleotide polymorphisms (SNPs) *STAT4* rs10181656, rs7574865, rs7601754, and rs10168266 were analyzed. The MS group consisted of 200 people: 88 males (44%) and 112 females (56%). The patients’ median age was 38 years (IQR = 15). The control group consisted of 200 people: 79 males (39.5%) and 121 females (60.5%). The control group’s median age was 33 (IQR = 21). No statistically significant differences between gender and age were found within the control and MS groups. Table 1 presents the subjects’ demographic information.

### 2.2. SNP Selection

Encoding a transcription factor belonging to the STAT family, the *STAT4* gene is found on human chromosome 2q32.3 [7]. The *STAT4* rs7574865, rs10181656, rs7601754, and rs10168266 were chosen for genotyping based on prior research on other autoimmune diseases. The SNP substitutions, SNP regions, chromosomal positions, and primer sequences are listed in Table 2.

The *STAT4* gene is thought to be linked to several autoimmune disorders; however, distinct susceptibility to the disease may result from different SNPs. The molecular mechanism of the *STAT4* gene’s involvement in the etiology of MS is still unknown because all mutations identified in this study are found in introns and do not directly affect STAT4 transcription or translation [20].

### 2.3. DNA Extraction and Genotyping

Each participant’s blood was collected into tubes with ethylenediaminetetraacetic acid (EDTA) Following the manufacturer’s instructions, a genomic DNA extraction kit based on silica-based membrane technology (Thermo Fisher Scientific, Vilnius, Lithuania) was used in the Laboratory of Ophthalmology, Neuroscience Institute, LUHS, to extract DNA. UV spectrophotometry (Agilent Technologies (Andover, MA, USA), Cary 60 UV-Vis) was used to determine the DNA concentrations and purity index in each blood sample as a ratio of absorbance 260/280 nm. Each sample displayed a purity index of 1.8 to 2.0. RT-PCR is a technique used to amplify and quantify DNA in real time, allowing for detecting and quantifying specific DNA sequences in a sample. The RT-PCR method comprised the following steps:

1. Primer design: Specific primers were designed to amplify the target DNA sequence. Primer sequences [VIC/FAM] are shown in Table 3.
2. Probe design: A fluorescent probe was designed to detect the amplified DNA sequence.
3. PCR reaction setup: The extracted DNA was mixed with the primers, probe, and other reagents needed for PCR amplification.
4. PCR amplification: The PCR reaction runs through cycles of denaturation, annealing, and extension, resulting in the exponential amplification of the target DNA sequence.
5. To ensure consistency of the genotyping process and accuracy of the results, a random sample comprising 5% (*n* = 20) of the total DNA samples was retested.
6. The data obtained from the RT-PCR were analyzed.

### 2.4. ELISA

Blood from peripheral vessels was collected to prepare serum. After 30 min of room temperature incubation, the blood samples were centrifuged. Following the pellet’s extraction, the serum was transferred into 2 mL tubes, refrigerated, and kept at −80 °C until analysis. The STAT4 serum levels of the control and MS patient groups were measured using the enzymatic immunoassay (ELISA) for human STAT4 (Human STAT4 ELISA Kit, Abbexa, Cambridge, UK) based on the conventional sandwich ELISA technique. The measurements were taken according to the manufacturer’s specifications. The optical density at 450 nm was measured using a microplate reader (Multiskan FC microplate photometer, Thermo Scientific, Waltham, MA, USA). The STAT4 serum levels were determined using the standard curve. The standard curve displayed a sensitivity of < 0.12 ng/mL and a range of 0.312–20 ng/mL.

### 2.5. Statistical Analysis

SPSS/W 29.0 (Statistical Package for the Social Sciences for Windows, Inc., Chicago, IL, USA) was the software used for the statistical analysis. The Kolmogorov–Smirnov test was used to determine whether the age was normally distributed. Continuous variables were shown as the median with the interquartile range (IQR) for data that were not normally distributed. To compare the two groups, the Mann–Whitney U test was performed. The chi-square (χ^2^) test examined the allele distributions, genotype, and gender differences between the MS and control groups. The categorical data were presented as absolute numbers with percentages. The binary logistic regression analysis was used to evaluate the effect of SNPs on MS. An odds ratio (OR) with a 95% confidence interval (CI) were provided for the results. Statistical genetic models were used to present the results of logistic regression. The best genetic model was identified using the Akaike information criterion (AIC). We evaluated four SNPs in the *STAT4* gene, and a two-tailed test with a value of less than 0.05 was considered statistically significant. The Bonferroni adjustment was used to modify the significance level for multiple comparisons (*p* = 0.0125 (0.05/4)). Serum STAT4 levels were compared between groups of MS patients and healthy individuals using the Mann–Whitney U test.

## 3. Results

### 3.1. STAT4 Variants Associations with MS Occurrence

After analyzing the genotypes and alleles of *STAT4* rs10181656, rs7574865, rs7601754, and rs10168266, we found that the *STAT4* rs7601754 AA genotype and the A allele were statistically significantly less frequent in MS patients compared to the control group (63.0% vs. 76.5%, *p* = 0.003, 79.0% vs. 87.0%, *p* = 0.003, respectively). No statistically significant differences were found between the distribution of genotypes and alleles of *STAT4* rs10181656, rs7574865, and rs10168266 in patients with MS and the control group (Table 4).

After analyzing the influence of MS occurence, binary logistic regression revealed that *STAT4* rs7601754 was statistically significantly associated with 1.9-fold increased odds of MS occurrence in the dominant model (OR = 1.912; 95% CI: 1.237–2.954; *p* = 0.004) and each G allele was associated with 1.7-fold increased odds of MS occurrence in the additive model (OR = 1.732; 95% CI: 1.193–2.516; *p* = 0.004), which were the best fit according to the AIC value, even after Bonferroni correction. The binary logistic regression analysis of the other SNPs showed no statistically significant results (Table 5).

### 3.2. STAT4 Variants Associations with MS Occurrence in Females

The pathogenesis of MS can be differentiated by gender; based on these data, we performed SNP analyses in males and females separately. The study revealed no statistically significant results after the Bonferroni correction (Table 6).

Furthermore, we used binary logistic regression analysis to assess how these SNPs affected females with MS. After the Bonferroni correction, no statistically significant results were found (Table 7).

### 3.3. STAT4 Variants Associations with MS Occurrence in Males

The analysis of *STAT4* rs10181656, rs7574865, rs7601754, and rs10168266 SNPs in males showed that, after strict Bonferroni correction, the rs7601754 AG genotype is more frequent in males with MS than in the control group (35.2% vs. 17.7%, *p* = 0.011) (Table 8).

After strict Bonferroni correction, binary logistic regression analysis in males revealed that only *STAT4* rs7601754 is associated with 2.5-fold increased odds of MS occurrence in males under the overdominant model (OR: 2.525; CI: 1.224–5.211; *p* = 0.012) (Table 9).

### 3.4. STAT4 Variants Associations with MS Occurrence in Patients Younger Than 37 Years

The genotype and allele distribution of *STAT4* genetic variant rs7601754 significantly differed between younger-than-37-year-old MS patients and the control group. However, when we applied Bonferroni’s corrected significance threshold, no statistically significant results were found (Table 10).

Binary logistic regression of *STAT4* rs10181656, rs7574865, rs7601754, and rs10168266 in younger than 37 years MS patients showed no statistically significant results (Table 11).

### 3.5. STAT4 Variants Associations with MS Occurrence in Patients Older Than 37 Years

The analysis showed no statistically significant results after the Bonferroni correction (Table 12).

We performed binary logistic regression analysis to evaluate the effects of these SNPs on MS patients older than 37 years. After Bonferroni corrections, no statistically significant results were found (Table 13).

### 3.6. STAT4 Serum Levels

**Figure 2:**
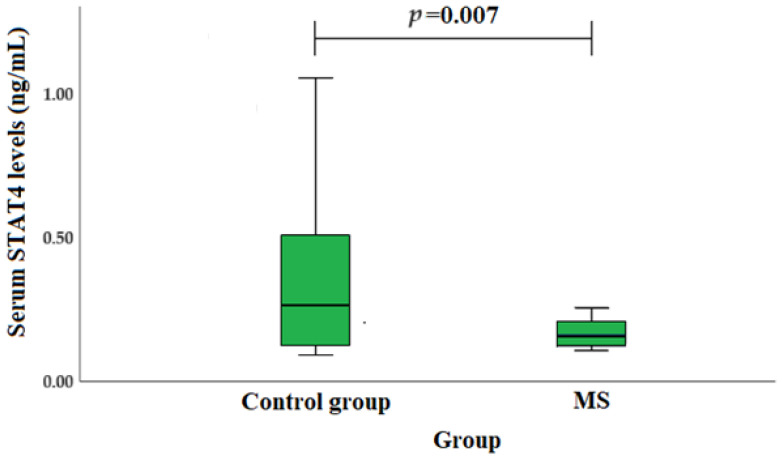
STAT4 concentrations in MS patients and healthy individuals.

Throughout the investigation, the blood serum concentration of STAT4 in the MS patient and healthy individual groups was measured. It was found that STAT4 serum concentration was statistically significantly lower in MS patients compared with the control group (median (IQR): 0.16 (0.09) vs. 0.26 (0.42), *p* = 0.007) (Figure 2).

## 4. Discussion

STAT4 is a transcription factor that plays a crucial role in developing autoimmune diseases [22]. It encodes an essential transcription factor that carries signals from specific cytokines linked to autoimmune disorders [8]. Since MS is an autoimmune disease, we looked for associations between *STAT4* SNPs, STAT4 serum levels, and MS. Even though *STAT4* has been linked to a variety of autoimmune disorders—neuromyelitis optica (NMO), systemic lupus erythematosus (SLE), rheumatoid arthritis (RA) systemic sclerosis (SS), MS [11,23,24,25,26]—this is, as far as we know, the first study to investigate the relationship between the *STAT4* (rs10181656, rs7574865, rs7601754, and rs10168266), STAT4 serum levels, and the occurrence of MS in the Lithuanian population. 

To our knowledge, there is only one study that has investigated an association between an *STAT4* variant and MS. Nageeb et al. hypothesized that *STAT4* rs7582694 gene polymorphism contributes to autoimmune diseases. The results showed that the CC genotype was statistically significantly more frequent in MS patients compared to the control group. Furthermore, the C allele was statistically significantly higher in patients with MS compared to controls [26].

The demyelinating condition known as NMO is a neurological disorder that matches many clinical characteristics with MS and fulfills all the requirements for an autoimmune origin [23]. Like MS, NMO causes episodes of optic neuritis and transverse myelitis. In both cases, a person’s immune system sees a healthy part of their body as a threat and attacks it. Shi et al. investigated the association between *STAT4* rs7601754 and NMO. The study showed that the G allele protects against NMO spectrum disorders (*p* = 0.006) [20]. Another autoimmune disease that can damage the CNS is SLE, characterized by various immunological abnormalities [24]. Several genetic studies have looked into the link between *STAT4* SNPs and SLE risk in different populations, but the results are inconsistent. A meta-analysis showed that *STAT4* rs7601754 and rs7574865 are significantly associated with SLE in European and African populations (*p* < 0.001) [27]. Another meta-analysis conducted by Wang and co-authors confirmed a strong association between the *STAT4* rs7574865 and rs10168266 and susceptibility to SLE (*p* < 0.001, *p* < 0.001, respectively). This study included 17,389 patients with SLE and 29,273 control subjects [28]. Ebrahimiyan et al. found that the *STAT4* rs7601754 A allele was significantly associated with a 0.679 lower susceptibility to SLE (OR = 0.679; 95% CI: 0.610–0.747, *p* < 0.001) [22]. Another study showed that the *STAT4* rs7574865 TT genotype and T allele are significant molecular risk markers for predicting susceptibility to SLE and that the GG genotype is a valuable marker against SLE risk [29]. Analysis of rs10168266 revealed that only the minor allele T was significantly associated with SLE in the Malaysian population (OR = 1.435; 95% CI: 1.143–1.802; *p* = 0.014) [30]. However, another study conducted by Salmaninejad et al. showed that both alleles A and G and the genotypes of rs7601754 did not show statistically significant differences between juvenile SLE patients and the control group [31].

As the studies show controversial results, we found that the A allele of rs7601754 is significantly associated with higher odds of MS occurrence according to the dominant model (OR = 1.912; 95% CI: 1.237–2.954; *p* = 0.004) and the additive model (OR = 1.732; 95% CI: 1.193–2.516; *p* = 0.004) after Bonferroni correction. In addition, the rs7601754 AG genotype is more common in males with MS than in the control group (35.2% vs. 17.7%, *p* = 0.011). Binary logistic regression analysis in males also revealed that only rs7601754 was associated with 2.5-fold increased odds of MS in males under the overdominant model (OR: 2.525; CI: 1.224–5.211; *p* = 0.012).

A great model for investigating how the immune system controls neural activity is MS. Accordingly, there is increasing evidence that pro-inflammatory mediators at high levels can seriously disrupt synaptic processes, neuronal excitability in general, and synaptic plasticity [32]. STAT4 is known for its regulatory role in proinflammatory signaling [33]. Additionally, STAT4 plays a critical role as a mediator in the development of inflammation in immunological-mediated diseases and protective immune responses. As a result of abrogated Th1 responses, STAT4-deficient mice are resistant to the development of Th1-mediated autoimmune diseases, including EAE, RA, colitis, myocarditis, and diabetes, because they produce a smaller amount of pro-inflammatory cytokines, such as tumor necrosis factor-alpha (TNF-α). [11]. A meta-analysis showed that the *STAT4* rs7574865 T allele was associated with RA in Europeans (OR = 1.300; 95% CI = 1.195–1.414; *p* < 0.001) [34]. Another study found a statistical association between rs10181656 and RA (*p* = 0.007) [35]. Furthermore, Hanan et al. found that patients carrying the T allele of rs7574865 have a high risk of RA and SLE compared to healthy controls (*p* < 0.001) [36]. It was also noticed that the rs7574865 T allele was statistically significantly associated with susceptibility to SS in the Spanish population (OR = 1.61; 95% CI: 1.29–1.99; *p* < 0.001) [25]. According to a study carried out by Zhang et al., the results showed a statistically significant association between the *STAT4* rs7601754 A allele and the risk of primary biliary cholangitis (OR = 1.35; 95% CI: 1.17–1.55; *p* < 0.001) [37]. Although various sources indicate associations of *STAT4* rs10181656, rs7574865, rs7601754, and rs10168266 with inflammatory and autoimmune diseases, in our study, only rs7601754 was statistically significantly associated with the occurrence of MS.

Inflammation depends on STAT, which controls the behavior of immune cells by facilitating the extracellular signaling of inflammatory mediators. Research shows that cytokines and growth factors can usually bind to their corresponding cell surface receptors to initiate an intracellular tyrosine kinase phosphorylation cascade. This cascade can be modified by kinases such as JAK2, which can alter immune responses, growth, and metabolic processes. Only a few studies have examined the association of STAT4 serum levels with disease risk. A study carried out by Zhang et al. revealed that the placenta of preeclampsia patients had statistically significantly higher STAT4 levels compared to normal late-term pregnant females [38]. It is also known that the increased systemic inflammatory response triggered by endotoxins is coordinated by excessive cytokine production. A study by Lentsch et al. showed that STAT4 is a vital regulator of the systemic inflammatory response to endotoxins. Mice lacking STAT4 are highly susceptible to lethal endotoxemia. These results indicate that STAT4 protects against endotoxin-induced death [39]. We found that serum STAT4 levels were statistically significantly lower in MS patients compared to the control group (median (IQR): 0.16 (0.09) vs. 0.26 (0.42), *p* = 0.007).

In conclusion, this was the first attempt to evaluate the association of *STAT4* SNPs and STAT4 serum levels with MS in the Lithuanian population. Although *STAT4* rs10181656, rs7574865, and rs10168266 have been associated with various types of autoimmune and inflammatory diseases, they were not considered as genetic factors contributing to MS in our patient group. Only *STAT4* rs7601754 is associated with MS and increases the disease occurence in the Lithuanian population. However, given the small number of patients in the case group of this study, further investigations with a sufficient sample size and in other populations, as well as an evaluation of different potential SNPs, will be helpful interpretations to reach a comprehensive conclusion about the role of *STAT4* in MS etiopathogenesis. The lack of association could be due to the small number of patients in the study group. Further studies with larger samples are needed to confirm these results and draw a conclusion.

## 5. Conclusions

In summary, the results of the present study show that *STAT4* rs7601754 increases the odds of MS occurrence. STAT4 serum levels were statistically significantly lower in MS patients compared to the control group. *STAT4* rs7601754 and STAT4 serum levels could be potential biomarkers associated with MS. Identifying *STAT4* variants and STAT4 serum levels’ impact on MS can help to identify personalized treatment strategies for individuals with MS. However, our results need to be verified in further studies.

## Figures and Tables

**Table 1:** Demographic characteristics of study groups.

Characteristics	Group		*p*-Value
	MS (*n* = 200)	Control Group (*n* = 200)	
Male, n (%)	88 (44.0)	79 (39.5)	0.417 ^1^
Female, n (%)	112 (56.0)	121 (60.5)	
Age, (years), median, (IQR)	38.0 (15)	33.0 (21)	0.143 ^2^

MS—multiple sclerosis; *p*-value—significance level (differences considered significant when *p* < 0.05). ^1^ Pearson chi-square; ^2^ Mann–Whitney U test.

**Table 2:** Information about *STAT4* SNPs used to amplify real-time polymerase chain reaction (RT-PCR) [20,21].

Rs Number	SNP Substitution	Region	Chromosome Position	HGVS Nomenclature
rs7574865	G>T	Intron 3	191,964,633	NC_000002.12:191099907T>G
rs10181656	C>G	Intron 3	191,969,879	NC_000002.12:191105152: G>C
rs7601754	G>A	Intron 4	191,940,45	NC_000002.12:191075724: G>A
rs10168266	C>T	Intron 5	191,935,804	NC_000002.12:191071077:C>T

SNP—single-nucleotide polymorphism; HGVS—Human Genome Variation Society.

**Table 3:** Primer sequences [VIC/FAM] of *STAT4* SNPs.

SNP	Primer Sequence
rs7574865	TATGAAAAGTTGGTGACCAAAATGT[G/T]ATAGTGGTTATCTTATTTCAGTGG
rs10181656	ACTAGCTGGAATCCAACTCTTCTCA[C/G]CCCTTGTACCACTACCCTCCTTTGT
rs7601754	CATGGGGGTGAAGAAAAGGAACTAC[G/A]CAAAGATGATACTAAGACCTTGATT
rs10168266	AGTAGTAGCTATTGACTACATGATA[C/T]ACTGTCTACCCACCCGTAGTAATAA

**Table 4:** Genotype and allele distribution of the *STAT4* variants in MS patients and the control groups.

Polymorphism	MS, *n* (%)	Control Group, *n* (%)	*p*-Value
*STAT4* rs10181656			
CC	122 (61.0)	117 (58.5)	0.307
CG	73 (36.5)	72 (36.0)	
GG	5 (2.5)	11 (5.5)	
Total	200 (100)	200 (100)	
Allele			
C	317 (79.25)	306 (76.5)	0.349
G	83 (20.75)	94 (23.5)	
*STAT4* rs7574865			
GG	125 (62.5)	118 (59.0)	0.214
GT	70 (35.0)	70 (35.0)	
TT	5 (2.5)	12 (6.0)	
Total	200 (100)	200 (100)	
Allele			
G	320 (80.0)	306 (76.5)	0.230
T	80 (20.0)	94 (23.5)	
*STAT4* rs7601754			
AA	**126 (63.0)** ^1^	**153 (76.5)** ^1^	**0.012**
AG	64 (32.0)	42 (21.0)	
GG	10 (5.0)	5 (2.5)	
Total	200 (100)	200 (100)	
Allele			
A	316 (79.0)	348 (87.0)	**0.003**
G	84 (21.0)	52 (13.0)	
*STAT4* rs10168266			
CC	134 (67.0)	133 (66.5)	0.441
CT	54 (27.0)	60 (30.0)	
TT	12 (6.0)	7 (3.5)	
Total	200 (100)	200 (100)	
Allele			
C	322 (80.5)	326 (81.5)	0.719
T	78 (19.5)	74 (18.5)	

^1^ AA vs. AG+GG *p* = 0.003; MS—multiple sclerosis; *p*-value—significance level. Bonferroni corrected the significance level when *p* < 0.0125 (0.05/4). Note: Significant results are indicated in bold.

**Table 5:** Analysis of *STAT4* variants using binary logistic regression in patients with MS and the control groups.

Model	Genotype/Allele	OR (95% CI)	*p*-Value	AIC
*STAT4* rs10181656				
Co-dominant	CG vs. CC	0.972 (0.644–1.469)	0.894	556.100
	GG vs. CC	0.436 (0.147–1.293)	0.134	
Dominant	CG+GG vs. CC	0.901 (0.604–1.344)	0.610	556.258
Recessive	GG vs. CC+CG	0.441 (0.150–1.292)	0.135	554.118
Overdominant	CG vs. CC+GG	1.022 (0.680–1.536)	0.917	556.507
Additive	G	0.845 (0.599–1.192)	0.336	555.591
*STAT4* rs7574865				
Co-dominant	GT vs. GG	0.944 (0.623–1.431)	0.786	555.346
	TT vs. GG	0.393 (0.134–1.150)	0.088	
Dominant	GT+TT vs. GG	0.863 (0.578–1.290)	0.474	556.004
Recessive	TT vs. GG+GT	0.402 (0.139–1.162)	0.092	553.420
Overdominant	GT vs. TT+GG	1.000 (0.663–1.508)	1.000	556.518
Additive	T	0.809 (0.574–1.139)	0.224	555.034
*STAT4* rs7601754				
Co-dominant	AG vs. AA	**1.850 (1.174–2.917)**	**0.008**	**549.602**
	AA vs. AA	2.429 (0.809–7.289)	0.114	
Dominant	AG+GG vs. AA	**1.912 (1.237–2.954)**	**0.004**	**547.825**
Recessive	GG vs. AA+AG	2.053 (0.689–6.117)	0.197	554.754
Overdominant	AG vs. AA+GG	1.770 (1.127–2.781)	0.013	550.271
Additive	G	**1.732 (1.193–2.516)**	**0.004**	**547.848**
*STAT4* rs10168266				
Co-dominant	CT vs. CC	0.893 (0.576–1.386)	0.614	556.867
	CC vs. CC	1.701 (0.650–4.455)	0.279	
Dominant	CT+TT vs. CC	0.978 (0.645–1.482)	0.915	556.506
Recessive	TT vs. CC+CT	1.760 (0.678–4.567)	0.245	555.121
Overdominant	CT vs. CC+TT	0.863 (0.559–1.333)	0.506	556.076
Additive	T	1.062 (0.755–1.494)	0.728	556.397

MS—multiple sclerosis; OR—odds ratio; AIC—Akaike information criterion; *p*-value—significance level. Bonferroni corrected the significance level when *p* < 0.0125 (0.05/4). Note: Significant results are indicated in bold.

**Table 6:** Genotype and allele distribution of the *STAT4* variants in females with MS and the control groups.

Polymorphism	MS, *n* (%)	Control Group, *n* (%)	*p*-Value
*STAT4* rs10181656			
CC	66 (58.9)	67 (55.4)	0.684
CG	42 (37.5)	47 (38.8)	
GG	4 (3.6)	7 (5.8)	
Total	112 (100)	121 (100)	
Allele			
C	174 (77.7)	181 (74.8)	0.465
G	50 (22.3)	61 (25.2)	
*STAT4* rs7574865			
GG	70 (62.5)	66 (54.5)	0.248
GT	39 (34.8)	47 (38.8)	
TT	3 (2.7)	8 (6.6)	
Total	112 (100)	121 (100)	
Allele			
G	179 (79.9)	179 (74.0)	0.129
T	45 (20.1)	63 (26.0)	
*STAT4* rs7601754			
AA	72 (64.3)	92 (76.0)	0.030
AG	33 (29.5)	28 (23.1)	
GG	7 (6.3)	1 (0.8)	
Total	112 (100)	121 (100)	
Allele			
A	177 (79.0)	212 (87.6)	0.013
G	47 (21.0)	30 (12.4)	
*STAT4* rs10168266			
CC	73 (65.2)	78 (64.5)	0.935
CT	37 (33.0)	40 (33.1)	
TT	2 (1.8)	3 (2.5)	
Total	112 (100)	121 (100)	
Allele			
C	183 (81.7)	196 (81.0)	0.845
T	41 (18.3)	46 (19.0)	

MS—multiple sclerosis; *p*-value—significance level. Bonferroni corrected the significance level when *p* < 0.0125 (0.05/4).

**Table 7:** Analysis of *STAT4* variants using binary logistic regression in females with MS and the control groups.

Model	Genotype/Allele	OR (95% CI)	*p*-Value	AIC
*STAT4* rs10181656				
Co-dominant	CG vs. CC	0.907 (0.530–1.553)	0.907	325.889
	GG vs. CC	0.580 (0.162–2.075)	0.580	
Dominant	CG+GG vs. CC	0.865 (0.514–1.454)	0.584	324.358
Recessive	GG vs. CC+CG	0.603 (0.172–2.119)	0.430	324.016
Overdominant	CG vs. CC+GG	0.945 (0.557–1.604)	0.833	324.614
Additive	G	0.845 (0.544–1.312)	0.453	324.094
*STAT4* rs7574865				
Co-dominant	GT vs. GG	0.782 (0.45501.345)	0.374	323.785
	TT vs. GG	0.354 (0.090–1.390)	0.137	
Dominant	GT+TT vs. GG	0.720 (0.426–1.216)	0.219	323.141
Recessive	TT vs. GG+GT	0.389 (0.101–1.504)	0.171	322.576
Overdominant	GT vs. TT+GG	0.841 (0.493–1.434)	0.525	324.255
Additive	T	0.704 (0.451–1.100)	0.123	322.247
*STAT4* rs7601754				
Co-dominant	AG vs. AA	1.506 (0.834–2.718)	0.174	319.089
	AA vs. AA	8.944 (1.076–74.358)	0.043	
Dominant	AG+GG vs. AA	1.762 (0.998–3.113)	0.051	320.800
Recessive	GG vs. AA+AG	8.000 (0.968–66.091)	0.054	318.944
Overdominant	AG vs. AA+GG	1.387 (0.772–2.493)	0.274	323.455
Additive	G	1.835 (1.116–3.017)	0.017	318.679
*STAT4* rs10168266				
Co-dominant	CT vs. CC	0.988 (0.571–1.712)	0.967	326.523
	CC vs. CC	0.712 (0.116–4.385)	0.715	
Dominant	CT+TT vs. CC	0.969 (0.566–1.660)	0.909	324.646
Recessive	TT vs. CC+CT	0.715 (0.117–4.361)	0.716	324.524
Overdominant	CT vs. CC+TT	0.999 (0.578–1.725)	0.997	324.659
Additive	T	0.950 (0.583–1.549)	0.838	324.617

MS—multiple sclerosis; OR—odds ratio; AIC—Akaike information criterion; *p*-value—significance level. Bonferroni corrected the significance level when *p* < 0.0125 (0.05/4).

**Table 8:** Genotype and allele distribution of the *STAT4* variants in males with MS and the control groups.

Polymorphism	MS, *n* (%)	Control Group, *n* (%)	*p*-Value
*STAT4* rs10181656			
CC	56 (63.6)	50 (63.3)	0.316
CG	31 (35.2)	25 (31.6)	
GG	1 (1.1)	4 (5.1)	
Total	88 (100)	79 (100)	
Allele			
C	143 (81.25)	125 (79.1)	0.625
G	33 (18.75)	33 (20.9)	
*STAT4* rs7574865			
GG	55 (62.5)	52 (65.8)	0.483
GT	31 (35.2)	23 (29.1)	
TT	2 (2.3)	4 (5.1)	
Total	88 (100)	79(100)	
Allele			
G	141 (80.1)	127 (80.4)	0.951
T	35 (19.9)	31 (19.6)	
*STAT4* rs7601754			
AA	54 (61.4)	61 (77.2)	0.038
AG	31 (35.2) ^1^	14 (17.7) ^1^	
GG	3 (3.4)	4 (5.1)	
Total	88 (100)	79 (100)	
Allele			
A	139 (79.0)	136 (86.1)	0.089
G	37 (21.0)	22 (13.9)	
*STAT4* rs10168266			
CC	61 (69.3)	55 (69.6)	0.266
CT	17 (19.3)	20 (25.3)	
TT	10 (11.4)	4 (5.1)	
Total	88 (100)	79 (100)	
Allele			
C	139 (79.0)	130 (82.3)	0.719
T	37 (21.0)	28 (17.7)	

^1^ AG vs. AA+GG *p* = 0.011. MS—multiple sclerosis; *p*-value—significance level. Bonferroni corrected the significance level when *p* < 0.0125 (0.05/4).

**Table 9:** Analysis of *STAT4* variants using binary logistic regression in males with MS and control groups.

Model	Genotype/Allele	OR (95% CI)	*p*-Value	AIC
*STAT4* rs10181656				
Co-dominant	CG vs. CC	1.107 (0.578–2.122)	0.759	232.600
	GG vs. CC	0.223 (0.024–2.064)	0.186	
Dominant	CG+GG vs. CC	0.985 (0.524–1.851)	0.963	233.024
Recessive	GG vs. CC+CG	0.216 (0.024–1.970)	0.174	230.694
Overdominant	CG vs. CC+GG	1.175 (0.616–2.239)	0.625	232.786
Additive	G	0.867 (0.397–1.511)	0.614	232.772
*STAT4* rs7574865				
Co-dominant	GT vs. GG	1.274 (0.659–2.464)	0.471	233.558
	TT vs. GG	0.473 (0.083–2.691)	0.398	
Dominant	GT+TT vs. GG	1.156 (0.613–2.179)	0.655	232.826
Recessive	TT vs. GG+GT	0.436 (0.078–2.448)	0.346	232.079
Overdominant	GT vs. TT+GG	1.324 (0.689–2.545)	0.400	232.313
Additive	T	1.017 (0.590–1.754)	0.951	233.022
*STAT4* rs7601754				
Co-dominant	AG vs. AA	2.501 (1.206–5.189)	0.014	228.357
	AA vs. AA	0.847 (0.181–3.956)	0.833	
Dominant	AG+GG vs. AA	2.134 (1.082–4.206)	0.029	228.081
Recessive	GG vs. AA+AG	0.662 (0.143–3.053)	0.597	232.742
Overdominant	AG vs. AA+GG	**2.525 (1.224–5.211)**	**0.012**	**226.402**
Additive	G	1.597 (0.907–2.812)	0.105	230.295
*STAT4* rs10168266				
Co-dominant	CT vs. CC	0.766 (0.365–1.610)	0.482	232.301
	CC vs. CC	2.254 (0.669–7.600)	0.190	
Dominant	CT+TT vs. CC	1.014 (0.524–1.962)	0.966	233.024
Recessive	TT vs. CC+CT	2.404 (0.723–7.998)	0.153	230.796
Overdominant	CT vs. CC+TT	0.706 (0.339–1.470)	0.353	232.158
Additive	T	1.178 (0.728–1.907)	0.504	232.576

MS—multiple sclerosis; OR—odds ratio; AIC—Akaike information criterion; *p*-value—significance level. Bonferroni corrected the significance level when *p* < 0.0125 (0.05/4). Note: Significant results are indicated in bold.

**Table 10:** Genotype and allele distribution of the *STAT4* variants in patients younger than 37 years with MS and the control groups.

Polymorphism	MS, *n* (%)	Control Group, *n* (%)	*p*-Value
*STAT4* rs10181656			
CC	58 (61.1)	62 (54.9)	0.302
CG	35 (36.8)	44 (38.9)	
GG	2 (2.1)	7 (6.2)	
Total	95 (100)	113 (100)	
Allele			
C	151 (79.5)	168 (74.3)	0.217
G	39 (20.5)	58 (25.7)	
*STAT4* rs7574865			
GG	60 (63.2)	64 (56.6)	0.217
GT	33 (34.7)	41 (36.3)	
TT	2 (2.1)	8 (7.1)	
Total	95 (100)	113 (100)	
Allele			
G	153 (80.5)	169 (74.8)	0.148
T	37 (19.5)	57 (25.2)	
*STAT4* rs7601754			
AA	61 (64.2)	87 (77.0)	0.127
AG	31 (32.6)	24 (21.2)	
GG	3 (3.2)	2 (1.8)	
Total	95 (100)	113 (100)	
Allele			
A	153 (80.5)	198 (87.6)	0.047
G	37 (19.5)	28 (10.4)	
*STAT4* rs10168266			
CC	64 (67.4)	74 (65.5)	0.741
CT	27 (28.4)	36 (31.9)	
TT	4 (4.2)	3 (2.7)	
Total	95 (100)	113 (100)	
Allele			
C	155 (81.6)	184 (81.4)	0.966
T	35 (18.4)	42 (18.6)	

MS—multiple sclerosis; *p*-value—significance level. Bonferroni corrected the significance level when *p* < 0.0125 (0.05/4).

**Table 11:** Analysis of *STAT4* variants using binary logistic regression in patients younger than 37 years with MS and control groups.

Model	Genotype/Allele	OR (95% CI)	*p*-Value	AIC
*STAT4* rs10181656				
Co-dominant	CG vs. CC	0.850 (0.481–1.504)	0.577	288.246
	GG vs. CC	0.305 (0.061–1.531)	0.149	
Dominant	CG+GG vs. CC	0.776 (0.445–1.350)	0.369	287.979
Recessive	GG vs. CC+CG	0.326 (0.066–1.606)	0.168	286.557
Overdominant	CG vs. CC+GG	0.915 (0.521–1.606)	0.756	288.693
Additive	G	0.733 (0.454–1.184)	0.204	287.153
*STAT4* rs7574865				
Co-dominant	GT vs. GG	0.859 (0.482–1.530)	0.605	287.499
	TT vs. GG	0.267 (0.054–1.306)	0.103	
Dominant	GT+TT vs. GG	0.762 (0.436–1.332)	0.340	287.876
Recessive	TT vs. GG+GT	0.282 (0.058–1.363)	0.115	285.767
Overdominant	GT vs. TT+GG	0.935 (0.528–1.654)	0.817	288.736
Additive	T	0.712 (0.443–1.145)	0.161	286.787
*STAT4* rs7601754				
Co-dominant	AG vs. AA	1.842 (0.986–3.443)	0.056	286.663
	AA vs. AA	2.139 (0.347–13.189)	0.413	
Dominant	AG+GG vs. AA	1.865 (1.017–3.421)	0.044	284.688
Recessive	GG vs. AA+AG	1.810 (0.296–11.063)	0.521	288.367
Overdominant	AG vs. AA+GG	1.796 (0.964–3.346)	0.065	285.353
Additive	G	1.721 (1.001–2.961)	0.050	284.844
*STAT4* rs10168266				
Co-dominant	CT vs. CC	0.867 (0.476–1.581)	0.642	288.190
	CC vs. CC	1.542 (0.333–7.147)	0.580	
Dominant	CT+TT vs. CC	0.919 (0.515–1.639)	0.775	288.708
Recessive	TT vs. CC+CT	1.612 (0.352–7.388)	0.539	288.407
Overdominant	CT vs. CC+TT	0.849 (0.468–1.541)	0.591	288.500
Additive	T	0.989 (0.601–1.627)	0.966	288.788

MS—multiple sclerosis; OR—odds ratio; AIC—Akaike information criterion; *p*-value—significance level. Bonferroni corrected the significance level when *p* < 0.0125 (0.05/4).

**Table 12:** Genotype and allele distribution of the *STAT4* variants in patients older than 37 years with MS and the control groups.

Polymorphism	MS, *n* (%)	Control Group, *n* (%)	*p*-Value
*STAT4* rs10181656			
CC	64 (61.0)	55 (63.2)	0.720
CG	38 (36.2)	28 (32.2)	
GG	3 (2.9)	4 (4.6)	
Total	105 (100)	87 (100)	
Allele			
C	166 (79.0)	138 (79.3)	0.950
G	44 (21.0)	36 (20.7)	
*STAT4* rs7574865			
GG	65 (61.9)	54 (62.1)	0.800
GT	37 (35.2)	29 (33.3)	
TT	3 (2.9)	4 (4.6)	
Total	105 (100)	87 (100)	
Allele			
G	167 (79.5)	137 (78.7)	0.850
T	43 (20.5)	37 (21.3)	
*STAT4* rs7601754			
AA	65 (61.9)	66 (75.9)	0.112
AG	33 (31.4)	18 (20.7)	
GG	7 (6.7)	3 (3.4)	
Total	105 (100)	87 (100)	
Allele			
A	163 (77.6)	150 (86.2)	0.031
G	47 (22.4)	24 (13.8)	
*STAT4* rs10168266			
CC	70 (66.7)	59 (67.8)	0.681
CT	27 (25.7)	24 (27.6)	
TT	8 (7.6)	4 (4.6)	
Total	105 (100)	87 (100)	
Allele			
C	167 (79.5)	142 (81.6)	0.608
T	43 (20.5)	32 (18.4)	

MS—multiple sclerosis; *p*-value—significance level. Bonferroni corrected the significance level when *p* < 0.0125 (0.05/4).

**Table 13:** Analysis of *STAT4* variants using binary logistic regression in older-than-37-years patients with MS and control groups.

Model	Genotype/Allele	OR (95% CI)	*p*-Value	AIC
*STAT4* rs10181656				
Co-dominant	CG vs. CC	1.166 (0.636–2.140)	0.619	267.823
	GG vs. CC	0.645 (0.138–3.006)	0.576	
Dominant	CG+GG vs. CC	1.101 (0.613–1.979)	0.747	266.375
Recessive	GG vs. CC+CG	0.610 (0.133–2.804)	0.526	266.070
Overdominant	CG vs. CC+GG	1.195 (0.655–2.179)	0.561	266.139
Additive	G	1.017 (0.613–1.686)	0.949	266.474
*STAT4* rs7574865				
Co-dominant	GT vs. GG	1.060 (0.579–1.942)	0.851	266.035
	TT vs. GG	0.623 (0.134–2.906)	0.547	
Dominant	GT+TT vs. GG	1.007 (0.56101.808)	0.981	266.478
Recessive	TT vs. GG+GT	0.610 (0.133–2.804)	0.526	266.070
Overdominant	GT vs. TT+GG	1.088 (0.598–1.981)	0.782	266.402
Additive	T	0.951 (0.574–1.576)	0.847	266.441
*STAT4* rs7601754				
Co-dominant	AG vs. AA	1.862 (0.954–3.633)	0.069	264.038
	AA vs. AA	2.369 (0.587–9.562)	0.226	
Dominant	AG+GG vs. AA	1.934 (1.031–3.630)	0.040	262.143
Recessive	GG vs. AA+AG	2.000 (0.501–7.978)	0.326	265.445
Overdominant	AG vs. AA+GG	1.757 (0.906–3.408)	0.095	263.627
Additive	G	1.705 (1.014–2.868)	0.044	262.205
*STAT4* rs10168266				
Co-dominant	CT vs. CC	0.948 (0.495–1.816)	0.873	267.694
	CC vs. CC	1.686 (0.483–5.879)	0.413	
Dominant	CT+TT vs. CC	1.054 (0.575–1.931)	0.866	266.450
Recessive	TT vs. CC+CT	1.711 (0.497–5.887)	0.394	265.719
Overdominant	CT vs. CC+TT	0.909 (0.378–1.727)	0.770	266.393
Additive	T	1.122 (0.698–1.804)	0.633	266.250

MS—multiple sclerosis; OR—odds ratio; AIC—Akaike information criterion; *p*-value—significance level. Bonferroni corrected the significance level when *p* < 0.0125 (0.05/4).

## Data Availability

The data presented in this study are available on request from the corresponding author due to ethical reasons.
